# Stool cultures show a lack of impact in the management of acute gastroenteritis for hospitalized patients in the Bronx, New York

**DOI:** 10.1186/s13099-020-00369-2

**Published:** 2020-06-22

**Authors:** Omar Fraij, Neva Castro, Luis A. de Leon Castro, Lawrence J. Brandt

**Affiliations:** 1 Division of Gastroenterology, Department of Medicine, Montefiore Medical Center, Albert Einstein College of Medicine, The Bronx, NY USA; 2 New York City Health and Hospitals Corporation (HHC), New York, NY USA

**Keywords:** Acute Gastroenteritis, Stool Cultures, Acute Diarrhea

## Abstract

**Background:**

Acute gastroenteritis (AGE) is diagnosed with a presentation of > 1 episode of vomiting and > 3 episodes of diarrhea in a 24-h period. Treatment is supportive, however, in severe cases antibacterial treatment may be indicated. Stool cultures can detect the responsible pathogenic bacteria and can guide antibiotic treatment, however, the indication for and efficacy of stool cultures is debatable. This study aimed to address the clinical utility of stool cultures in patients diagnosed with AGE.

**Methods:**

A retrospective, multicenter study was performed in patients admitted for AGE from 2012 to 2014. Patient charts were obtained through hospital software using ICD-9 codes for AGE. Inclusion criteria was a documented diagnosis of AGE, age of 18 years or older, symptoms of both upper GI symptoms of abdominal pain and/or nausea and lower GI symptoms of diarrhea. Patients were classified into two main groups, those in whom (1) stool culture was obtained and (2) those in whom stool culture was not performed. Clinical features and outcomes were compared between groups. The diagnostic yield of stool cultures was assessed. All analysis were conducted using the Statistical Package for Social Science (SPSS).

**Results:**

Of 2479 patient charts reviewed, 342 met the above criteria for AGE. 119 patients (34.8%) had stool cultures collected and 223 (65.2%) did not. Demographics, clinical features and serologic lab values are shown in Table 1. Of the 119 stool cultures performed, only 4% (n = 5) yielded growth of pathogenic bacteria (2 Pseudomonas spp, 2 Campylobacter spp, 1 Salmonella spp). The group who underwent stool culture had a higher percentage of patients with fevers (26% vs 13%,*p* < 0.003) and longer hospital length of stay (3.15 vs 2.28 days, *p *< 0.001) compared to the group that did not undergo stool cultures.

**Conclusion:**

Stool cultures are commonly ordered when AGE is suspected. In our cohort, stool culture had a very low yield of detecting an underlying pathogen. Although patients who had stool cultures obtained were more likely to be febrile and to have a longer length of hospital stay than were those who did not have stool cultures, for the vast majority of patients, stool culture played little to no role in patient management. Further studies are needed to which patients benefit most from undergoing stool culture.

## Introduction

Acute Gastroenteritis (AGE) of the most common diseases throughout the world. In the USA 76 million cases of foodborne disease occur each year, forcing 1 in 8 adults to visit the Emergency Department in their lifetime yearly and causing over 500,000 hospitalizations annually [1–3]. In general the common cause of AGE is a virus followed by bacteria and parasites however specific pathogens varies throughout geographical regions [1, 4, 5, 18]. Infection of the intestinal tract results in abdominal pain, fever, nausea, vomiting, and diarrhea. Unlike colitis in which computed tomography (CT) can aide in diagnosis [6], AGE is diagnosed clinically. Presentations vary, however, agreed upon clinical criteria for the diagnosis of AGE include > 1 episode of vomiting and > 3 episodes of diarrhea in a 24-h period [1, 7]. Treatment is most typically supportive with a focus on hydration, however, in severe case antibacterial treatment is indicated. Guidelines of the American College of Gastroenterology recommend diagnostic studies of stool, if available, in cases of dysentery, moderate-to-severe disease, and symptoms lasting > 7 days to clarify the etiology of the patient’s illness and enable specific directed therapy [8]. A myriad of such diagnostic techniques for stool samples include bacterial stool culture, microscopy, stool antigen testing, PCR, and stool leukocytes. Bacterial stool culture workup consists of examining culture media for colonies that display phenotypic properties consistent with those of enteric pathogens, these colonies are then are further screened using select biochemical tests to identify a wide spectrum of bacteria with the more common bacterial species being Salmonella, Shigella, Campylobacter, or *E. coli* [9]. Stool culture was thought to be able to help confirm the diagnosis of AGE and potentially clarify whether antimicrobial therapy is indicated [7]. Despite this availability, there have been prior studies that have questioned the efficacy of this costly and often times burdensome analysis [7, 10–12]. In < 20% of patients admitted to the hospital with AGE ultimately have a specific pathogen identified as etiologic [13]. In another study of > 30,000 patients hospitalized with diarrhea, < 6% had an bacterial pathogen identified by stool studies [14]. There have been previous studies to evaluate the diagnostic efficacy of stool culture, however, none have compared the effects on medical outcome of having stool cultures versus not having stool culture. In this study we aimed to determine the utility of stool culture and whether this test impacts management of AGE.

## Methods

A retrospective record review was done of patients admitted to the Montefiore Medical Center and Jacobi Medical Center from 2012 to 2014 with a diagnosis of AGE. All studies and data were conducted with the approval of the Institutional Review Board at Albert Einstein College of Medicine. Montefiore Medical Center is a 1498 bed university hospital for the Albert Einstein College of Medicine and Jacobi Medical Center is a 776 bed city hospital in the Bronx, NY, also is affiliated with Albert Einstein College of Medicine. ICD-9 codes 009.0 and 558.9 for infectious and non-infectious gastroenteritis respectively were used to obtain patient charts using Clinical Looking Glass (CLG), the patented analytics software program used at Montefiore Medical Center. Inclusion criteria for patients with AGE were age of 18 years or older, and symptoms of AGE, i.e., abdominal pain and/or nausea and diarrhea. Patients with ICU stay were excluded to reduce confounding factors. Patients who met AGE criteria were divided into two cohorts: those in whom stool cultures were obtained; and those in whom no stool cultures were obtained. Data were compared between groups. Demographic data recorded included gender, age, and race. Socioeconomic data regarding private versus private medical insurance was recorded. Clinical features recorded included fever (temperature > 38.0 °C), and white blood cell (WBC) count.

Antibiotic use rate and amount was recorded by reviewing the medical administration record (MAR) and the discharge medical reconciliation list. Defined daily dose (DDD) method, which is the average maintenance dose per day for a drug used, was used to quantify antibiotics and present them in units for comparative purposes. Computed tomography (CT scan) results were also recorded when present. We also recorded the hospital length of stay and readmission rate to assess for effectiveness of the medical treatment.

Statistical Analyses: All analyses were conducted using the Statistical Package for Social Science (SPSS). Group comparisons were performed using nonparametric Mann–Whitney rank sum test for continuous variables and Pearson’s chi-squared test for categorical variables. Fisher exact test was used for binary variables. To assess univariate associations between a positive stool culture and clinical features, logistic regression analysis was performed. All p values were two-sided and considered statistically significant if *p *< 0.05.

## Results

**Fig. 1:**
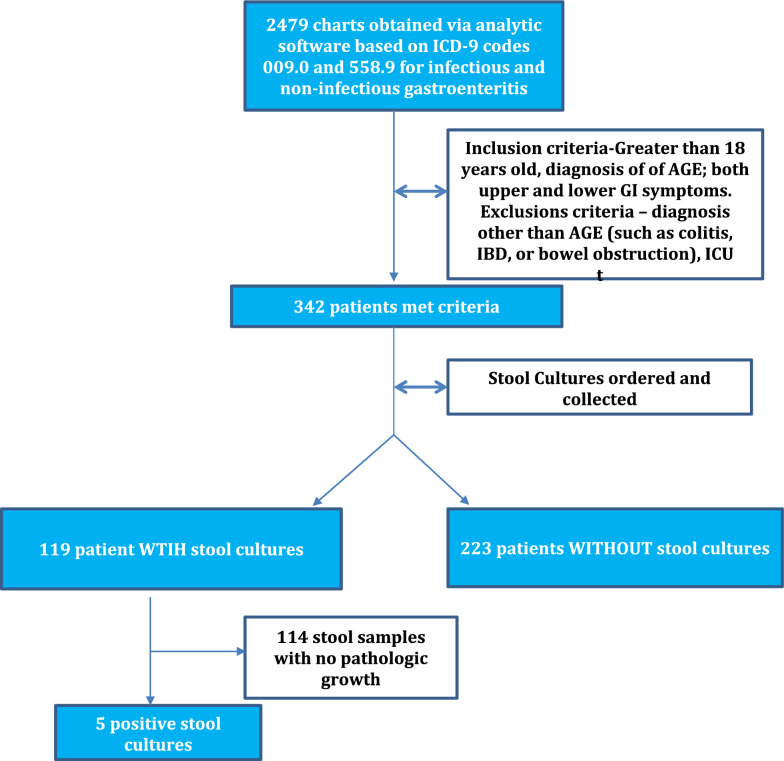
Cohort assignment

During the study period, there was a total of 2479 patient charts retrieved using the ICD 9 codes cited above. 342 adult patients met the study inclusion criteria (Fig. 1) of which 283 (82.7%) were from Montefiore Medical Center and 59(17.3%) were from Jacobi Medical Center. Of the 342 patients with admissions for AGE, 230 (67.3%) had stool cultures ordered and 119 of the 230(51.7%) were actually collected. The stool culture-cohort (SC-cohort) of (n = 119) was compared with the non-stool culture-cohort (NSC-cohort) (n = 223). Demographics, insurance type, clinical features, presence of CT scan, and WBC lab values are shown in Table 1. The median ages for both the SC- and NSC cohorts (55 years vs 58 years, respectively), were similar. The majority of the patients were female and had public insurance with the SC-cohort showing 68.9% female and 83% public insurance versus the NSC-cohort showing 72.9% female and 87% publicly insured, this was not statistically significant. CT scans were being ordered at a rate of 58% with the SC-cohort showing enteritis at rate of 33% versus the NSC-cohort enteritis rate of 53%, this was not statistically significant. There was a non-statistically significant higher DDD median in the SC-cohort than in the NSC-cohort, (6.91 vs 4.00, p = 0.347). Admission WBC (9.5 vs 9.0) and discharge WBC (6.2 vs 6.7) was similar in both cohorts and not statistically significant. The SC-cohort had a higher percentage of subjects with fever compared with subjects in the NSC-group (26% vs 13%, *p* < 0.003). Additionally, hospital length of stay was longer in the SC-cohort (3.15 vs 2.28 days, *p* < 0.001). Readmission rate within 30 days was higher in SC-cohort compared with the NSC-cohort (14.3% vs 8.5%, *p* = 0.098). Of the 119 stool cultures, only 5 (4.2%) yielded pathological bacterial growth (Table 2). Of these 5 patients who had positive stool cultures 3 were treated supportively with no antibiotic administration with resolution of disease.

**Table 1:** Demographics and outcomes of AGE

	No culture	Culture	Total	*p*
	(n = 223)	(n = 119)	(n = 342)	
Demographics				
Age (years)	58 [38–72.5]	55 [39.5–71.5]	57 [38–72]	0.99
Gender				0.47
Female	162 (72.6)	82 (68.9)	244 (71.3)	
Male	61 (27.4)	37 (31.1)	98 (28.7)	
Race				0.43
White	30 (13.7)	15 (12.7)	45 (13.4)	
Black	77 (35.2)	32 (27.1)	109 (32.3)	
Hispanic	97 (44.3)	61 (51.7)	158 (46.9)	
Other	15 (6.8)	10 (8.5)	25 (7.4)	
Insurance type				
Private	29 (13%)	20 (17%)	49 (14%)	0.34
Public	194 (87%)	99 (83%)	293 (86%)	
Hospital details				
Admission WBC^a^	9 [6.6–12.2]	9.5 [7.65–12.75]	9.3 [6.9–12.4]	0.14
Last WBC^a^	6.7 [5.2–8.7]	6.2 [4.9–8]	6.5 [5.1–8.3]	0.12
Antibiotics	77 (52%)	62 (35%)	139 (41%)	
DDD^a^	4 [2–14]	6.91 [2–14]	5 [2–14]	0.35
Fever^a^	29 (13)	31 (26.1)	60 (17.5)	*<0.01*
CT completed	81 (68%)	118 (53%)	199 (58%)	
CT with enteritis	32/81 (53%)	39/118 (33%)	71/199 (35%)	0.35
Outcome				
LOS (Days)^a^	2.28 [1.73–3.42]	3.15 [2.22–4.67]	2.64 [1.86–3.94]	*<0.01*
Readmission^a^	19 (8.5)	17 (14.3)	36 (10.5)	0.09

Values are median [interquartile range] or n (%)

^a^Definitions: (1) WBC: white blood cell count; (2) DDD: WHO’s defined daily dose; (3) Fever: temperature > 100.4°F; (4) LOS: Length of stay in days; (5) Readmission: patient admission to a hospital within 30 days after being discharged

**Table 2:** Positive stool cultures

Bacterial specie	Fever	Leukocytosis	CT scan	Antibiotic
Pseudomonas	No	Yes	Enteritis	None
Pseudomonas	No	Yes	Negative	Yes
Salmonella	Yes; 38.7 °C	No	Negative	Yes
Campylobacter	Yes; 39.3 °C	Yes	No CT scan	None
Campylobacter	Yes; 39.3 °C	No	Negative	None

## Discussion

Medical care is an evolving discipline with an intrinsic desire to improve efficacy and outcome. This study demonstrates that stool culture is a routinely ordered laboratory test despite its adding little clinical value. Based on our data, clinicians ordered stool cultures 67% of the time in AGE admissions which were obtained at a rate of 52%. The reasons for the low rate of stool specimen collection is not obvious, however, is likely attributable to the burdensome and unpleasant process of collecting a stool specimen or improvement of diarrhea in patients who clinically improved before the time of collection. As a diagnostic tool, the low rate of stool culture positivity expresses a low index of clinical value. In our study 4% of the stool cultures (Table 2) were positive which is similar to what has been observed in previous studies [10, 11, 15, 16]. Previous studies of AGE for adults in developed countries have shown Salmonella, Campylobacter, Yersinia, enterohemorrhagic *E. coli*, Vibrio species to be the more common bacterial pathogens [1, 7, 11]. In our SC-cohort, three stool samples yielded Salmonella and Campylobacter species which is consistent with a previous study of adult AGE patients in a hospital setting in the United States [1]. The finding of Pseudomonas specie in two of the five stool cultures was surprising as this is not considered a cause of AGE [1, 16–18]. Pseudomonas as has been known to colonize the gastrointestinal tract particularly in patients who have had bowel surgery however neither of the two patients in our study had these comorbidities nor were they immunocompromised [31]. Based on the stringent criteria in our study to only include patients who had acute gastroenteritis we believe the implication of Pseudomonas species as a cause of AGE should be further investigated. The low percentage of positive stool cultures in our study and other previous studies may also have to do with the limited range of bacterial pathogens cultures in standard medical laboratories [9]. A major limitation in stool cultures is that there are commonly nonbacterial causes of AGE. Several studies have shown the most common cause of AGE in adults in developed countries is norovirus which is diagnosed via PCR [1, 4]. Also parasites such as Giardia and Cryptosporidium species, which require wet mount with staining for identification, have been found to be the causative organism in AGE in other studies in similar settings to ours [1, 4].

The guidelines of the American College of Gastroenterology for management of diarrhea recommend that stool cultures in adults are indicated most appropriate in the presence of severe diarrhea, temperature > 38.5 °C (orally), passage of bloody stools, or diarrhea lasting more than 7 days [8]. These guidelines are designed to be lenient and one study showed the majority of stool culture orders meeting these criteria, despite low efficacy [7]. Our study did show that individuals with fever were twice as likely to have stool cultures ordered than were non-febrile individuals and that 3 of 5 stool cultures were positive when ordered in patients with a temperature of > 38.5 °C; this supports our observation that stool cultures are more valuable in patients with fever. In our study 40% of all AGE admissions were treated with antibiotics with more patients receiving antibiotics in the SC- cohort. Stool cultures require 24–72 h for completion which leads to antibiotics, if used, being administered prior to knowing the results [15]. To assess the effect of stool cultures on antibiotic use we calculated the DDD between the two groups, which showed that antibiotic use is higher in the SC-cohort; there was no clear trend to suggest that antibiotics use was reduced or tailored by using stool cultures. Our data also showed that individuals who had stool cultures on admission had a longer length of hospital stay, suggesting that patients who had stool cultures were clinically more symptomatic than those without stool studies. From our observations, it appeared that when stool cultures are ordered, clinicians are more likely also to order antibiotics with a plan to adjust or discontinue them after the results of stool culture becomes available. A similar strategy is often used with blood cultures in septic shock, however, in the case of AGE this will lead to antibiotic overuse; such overuse of antibiotics can cause more harm as it can lead to eradication of normal flora, antibiotic resistance, and super infection, e.g., with C. difficile [20–22].

Stool cultures require laboratory reagents and technical time. Because of its low yield in AGE, the estimated cost of $952 to $1,200 per positive culture makes it among the costliest laboratory diagnostics available [17, 23–25]. Moreover, by the time the stool culture result is available, the vast majority of patients have recovered which has prompted a strict “3-day rule” in some European countries to detect pathogenic bacteria and reduce the total numbers of stool cultures performed [7, 26, 27]. The “3-day rule” recommends that stool be collected for community-acquired diarrhea only if the onset of diarrhea is ≤ 72 h after admission; in nosocomial diarrhea, stool is cultured if the onset is > 72 h after admission with at least one of the following criteria: age ≥ 65 years with a preexisting comorbidity, HIV infection, neutropenia, or if there is a suspected nosocomial outbreak [11, 17]. Stool cultures may be indicated if there is a clinical and/or epidemiological suspicion of Vibrio cholerae, particularly in outbreaks/epidemics [28, 14]. Our study also showed CT scans were ordered in majority of patients presenting with AGE with no correlation to stool culture pathogen; findings of AGE on CT scan are nonspecific and CT does not play a major diagnostic role in detection or differential diagnosis [6]. Although our patient population had an average age of 57 years old, there is no standard screening protocol for patients in any age group with GI symptoms requiring a CT scan to rule out other etiologies such as malignancy. The finding that CT scans were ordered 58% of time suggests more focus dedicated to patient history and physical exam would likely reduce the need for this potentially harmful and costly modality.

In patients with AGE, management should be focused on symptomatic treatment, rehydration, prevention in spread of infection by maintenance of scrupulous personal hygiene and use of antibiotics only in indicated cases [4, 23, 24, 29]. Considering the costs and time required to obtain stool culture and its low diagnostic yield, there should be great reservation in ordering stool culture in patients suspected of having AGE. Advances in technology that will permit rapid pathogen-specific diagnostic testing, e.g., based on gene testing by PCR for bacteria, viruses, and parasites could reduce excessive and inappropriate use of antibiotics and identify sources of outbreaks including contaminated water or food [23, 29]. Some that are available include non-culture detection methods using enzyme immunoassays and molecular testing by syndromic panels which are often used when screening for* C*.* difficile* [9].

Our study showed that the majority of the patients admitted for AGE were female (71.3%) which is consistent with previous studies of similar settings [1, 30]. We also found that the vast majority of our patients were publicly insured (86%) which may suggest that a lack of outpatient access to healthcare may increase utilization of hospital care as has been evaluated in other studies however it is important to note that the majority of the population in Bronx, NY is publicly insured [31].

Our study was limited by its retrospective nature. The clinical features obtained were from during the admission period only and no data was analyzed after discharge. The low rate of bacterial growth in stool cultures might also reflect that less common bacterial pathogens may not have been tested for such as Providencia alcalifaciens, Escherichia albertii or Edwardsiella tarda [9]. In conclusion, our study was able to show that stool cultures remain a routine study ordered in the management of AGE. There are clear benefits to stool culture in febrile patients with severe disease that can dramatically improve patient care which should not be overlooked, however, more restraint in its use should be used to appropriately utilize costly resources.

## Data Availability

All data generated or analyzed during this study are included in this submission, attached under supplementary material.
